# Sustainability of top-performance athletes’ mental health and career retirement support services: European major sports organizations’ perspective

**DOI:** 10.3389/fspor.2026.1722556

**Published:** 2026-04-01

**Authors:** Flavia Guidotti, Francesca Di Rocco, Cristian Romagnoli, Simone Ciaccioni, Sabrina Demarie, Laura Capranica, Elvira Padua

**Affiliations:** 1 Department of Human Sciences and Promotion of the Quality of Life, San Raffaele Open University of Rome, Rome, Italy; 2 Department of Movement, Human and Health Sciences, University of Rome “Foro Italico”, Rome, Italy

**Keywords:** career retirement, career support, elite athletes, mental health support, survey

## Abstract

**Introduction:**

The transition out of elite sports presents a complex array of psychological, financial, career, and social challenges requiring tailored and sustainable support services for elite athletes. Thus, this study explored policies, programs, and practices surrounding elite athletes' mental health, career transitions, and retirement support services.

**Methods:**

A tailored semi-structured survey was administered to major sports organizations to assess typology, modality, funding, and perceived challenges of services delivery to elite athletes.

**Results:**

Respondent organizations (*n* = 17, mostly National Olympic Committees) highlighted issues in ensuring athletes adequate support and a fragmented approach in services implementation and delivery. Services are offered mainly to medalists in international competitions. Access to external professionals (34%), awareness training (20%), and in-house psychologists (17%) resulted the most cited mental health support services. However, the sustainability and quality of services are affected by a lack of funding (41%) and different assessment methods. For career transitions and additional services, educational workshops (29%), career counselling (19%), nutrition (21%), financial (19%), social (19%), and travel/logistics (19%) support resulted the most cited, meeting athletes' practical needs related to performance and well-being through internal organizational support networks (40%) and coach/staff referrals (33%).

**Discussions:**

Results highlighted the absence of regulatory frameworks for career assistance and temporary initiatives/activities or informal support as the common practice. Multi-faceted programs and comprehensive, athlete-centered systems are needed to foster a sustainable and effective support for elite athletes.

## 1 Introduction

The impact of sport on sustainability has been explored across multiple dimensions and sectors, ranging from the fight against climate change, over-consumption, economic exploitation, and social disadvantage (1). This growing attention to sustainability as part of sports ethics has led to the consideration of the whole sports and physical activity sectors from both a policy implementation perspective and a proactive approach to elicit a real and concrete impact in society, with the goal of promoting adaptability and capacity to function effectively in the future. In particular, sport can contribute to advancing the 2030 Agenda for Sustainable Development and its Sustainable Development Goals (2) through interventions across multiple areas and dimensions. Inclusive sport policies for marginalized groups, and the empowerment of individuals and communities represent key areas of implementation. This could be achieved through the promotion of quality education, lifelong learning, health and well-being, and access to employment in and through sport serving as key pillars for reaching the strategic goals (3–5). In this respect, sustainability development in sport embeds key elements for fair and equitable societies (e.g., integrity, equality, honesty, excellence, commitment, courage, team spirit, respect for rules and laws, respect for the environment, respect for self and others and a spirit of community, tolerance and solidarity), representing both issues requiring policy implementation and opportunities for a more sustainable sport sector.

Article 9 of the European Sport Charter (3) states that “the principle of sustainability in sport requires all activities to be economically, socially and environmentally sustainable. When planning, implementing and evaluating their activities, organizers of sports activities and events should pay due consideration to sustainability, be it economic, social or environmental”. From a social perspective, this highlights the need to emphasize good governance in sport (6), and to ensure the active engagement of all stakeholders in developing and integrating practices that uphold human rights, ethics, and integrity. In broader sense, this includes the protection of elite athletes’ rights, ensuring that “the human rights of athletes are respected, protected and promoted” (3).

Within this framework, good governance in sport is essential to ensure that organizations fulfill their responsibilities (6). Deficiencies in governance can undermine their capacity to prevent and address risks, generating uncertainty, unsafe conditions, and unfairness in elite athletes’ working environments and in their future opportunities beyond sport. Consequently, major sports organizations are required to integrate and implement principles of good governance into their policies and practices, particularly regarding the safeguarding, protection, and support of high-performance athletes and teams (7, 8). However, policy implementation cannot be separated from the national socio-cultural and organizational context (8). Thus, strategies and actions towards the promotion and protection elite athletes’ rights require meaningful and consistent implementation in light of both international standards (such as recommendations and policies delivered from major umbrella organizations (9, 10) and the internal (e.g., national, organizational) context and operating capacity.

Over the past 20 years, sports scholars have increasingly drawn on sustainability to question whether elite sport can be sustained over time, especially in considering its competition logic, precarity, and potential negative repercussions on several life aspects due to a consistent and prevalent sport involvement in active-career years. In particular, from the seminal studies of Loland (11, 12) and Lawson (13), an anthropocentric view emerged defining sustainability as “development that should secure future human generations the same possibilities to satisfy basic needs as our own”. From this perspective, sustainability depends on a balanced relationship among sociocultural, ecological, and economic systems that supports long-term human flourishing, such as in elite sports careers. Indeed, it has been argued that Olympic sport cannot persist indefinitely due to prevalent record-breaking logic confronting with human biological limits. This would demand increasingly extreme training programs and sport-related technological development for limits overcoming (12), which could result unstainable in the long-term. In this framework, a shift in emphasis from performance-oriented logics towards broader access and equal opportunity in participation in sport has been suggested as a strategy to foster sustainability in elite sports careers. Such approach should secure sustainable social relations, prioritize athletes’ physical and mental health, and enhance their future opportunities as citizens, outside their elite sport career journey. Hence, sustainability in elite sport career could be defined as a “sequence of career experiences reflected through a variety of patterns of continuity over time, thereby crossing several social spaces, characterized by individual agency, herewith providing meaning to the individual” (14). In this framework, a positive career transition could contribute to career sustainability, based on athletes’ sports, personal, phyco-social, academic, and vocational development, by building resources and skills for their lives after sports (15). This would also require context-specific education and development of elite athletes’ main stakeholders (e.g., sport governing bodies, clubs, managers, and coaches) to implement sustainable structures for long-term growth (16).

The transition from elite sport to post-athletic life is widely acknowledged as one of the most complex phases of an athlete's career (17), often jeopardizing the sustainability of athlete support systems across different career phases (18, 19). In particular, retirement from sport should be considered and managed as a multifactorial and longitudinal process involving psychological, social, financial, and vocational adjustments (20–22), encompassing identity reconstruction, transfer of sport-acquired skills, reintegration into society, and entry into the labor market. This requires proactive coping strategies and strategic career planning (23, 24), which could be burdened in case adequate support is not available. Elite athletic retirement may lead to mental health disorders, financial instability, unemployment, and social isolation, especially in cases of forced or sudden retirement such as injuries and deselection (20, 24–26), ultimately compromising both athlete well-being and the effectiveness and sustainability of elite sport systems in charge of safeguarding athletes’ rights and welfare. In fact, athletes often struggle to find meaning beyond their sport, especially during abrupt retirements, with elevated risks of depression, anxiety, substance misuse, and other mental health disorders (20, 23). Hence, the International Olympic Committee (IOC) took action and issued a Consensus Statement on Mental Health in Elite Athletes (27) and launched a specific Action Plan (28), advocating multidimensional strategies to normalize help-seeking (e.g., enhance mental health literacy, early screening, monitoring, availability of professional care) and emphasizing cultural integration of mental health protection within sport organizations (27, 29, 30) by establishing adequate services at national level.

Consensus statements and position stands on elite athletes’ mental health and career transitions (18, 31–34) call for national strategic implementation. Scientific evidence extensively highlighted the importance of structured, multi-dimensional and multi-stakeholder strategies to secure athlete welfare as a core component of sustainable sport development, with planned retirement trajectories, dual career pathways [e.g., combining elite sport demands with academic education and/or vocational training (35, 36)], career counselling services, and targeted mental health services emerging as effective protective factors against adverse career retirement outcomes [e.g., (19, 21, 23, 37–50)]. In particular, dual career strategies enable athletes to cultivate identities beyond their sport activity, thereby reducing the risks associated with the lack of occupational opportunities upon retirement, promoting smoother career transitions, and building stronger social and professional networks (51). Tailored mental health services including counseling, peer support, and discreet, accessible, and culturally appropriate assistance are crucial, although stigma often thwarts athletes accessing care and support. Embedding structured educational and career development programs (e.g., career counseling, vocational training, financial literacy, job placement services, mentorship, professional networking) within sport environments can also mitigate anxiety, depression, and financial insecurity during retirement, facilitating the integration into new personal and professional roles. Thus, structured transition programs and holistic support systems should be prioritized by sport organizations at all levels (52), recognizing and protecting elite athletes’ rights and well-being, and the transferability of their human and social capital to the broader society at the end of their sporting career.

The European Parliament (53) emphasized the protection of athletes’ fundamental rights, including fair work, decent conditions, and occupational safety, reframing retirement as an empowering career stage. In this framework, the role of major sport organizations and National Olympic Committees (NOCs) is crucial. Beyond preparing athletes for competition, these institutions bear responsibility for safeguarding holistic development across the athletic lifecycle. Initiatives such as the IOC's Athlete365 Career + program (17) provide education, training, and career transition services through workshops, online resources, and mentorship. Expanding such support across all levels and disciplines is vital for equity and sustainability. Hence, several organizations are embedding athlete career initiatives into their governance frameworks and concrete actions (54, 55), linking welfare to organizational sustainability. A necessary mention should be made regarding the different policy frameworks and career assistance models in place (55, 56), that significantly impact national action plans in achieving tangible outcomes. In fact, previous early literature in this field has highlighted different policy approaches in place within European countries in providing educational and career assistance services to elite athletes (56), ranging from structured and State-funded systems to “Laisser-Faire” modalities. Similarly, the study of Hong (55) providing a comprehensive analysis of career assistance services (e.g., pursue of sports excellence, development of life/transferable skills, achievement of a balance between athletic careers and non-athletic activities, dual careers, career transitions and retirement) for high-performance athletes across different sport organizations and countries highlighted potential disparities and gaps in services provision and reach. To note, most of the career assistance programs are dedicated to high-profile athletes and/or medalists. Therefore, athletes in the process of acquiring the high-level status necessary for eligibility to access the provided services are particularly at risk, lacking access to resources and encountering limited opportunities. Finally, it should be considered that athletes of different sports, countries, and stages of their careers might present different needs and difficulties, making the overall career assistance programs and strategies very intricated. Therefore, inclusivity and diversity of services provided by sport organizations for ensuring equal opportunities for high-performance athletes are still uneven (55), whereas reliance on limited financial resources constrain the provision of a consistent support in many settings (21). Ideally, sustainable transition frameworks should embed mental health, education, vocational training, career counseling, and financial planning throughout the athletic lifecycle rather than concentrating support at retirement. Such approaches align with the Sustainable Development Goals (4), reinforcing the role of sport in advancing societal sustainability. However, embedding athletes’ retirement within a sustainability paradigm stresses the need to prioritize holistic frameworks, cross-sectoral involvement, stakeholders’ engagement (e.g., NOCs, sports federations, athlete commissions, educators, healthcare providers, and policymakers), systemic equity, accountability, transparency, as well as resource allocation and durability. Despite the documented widespread recent progress in organizational policies and initiatives to enhance elite athletes’ welfare (55), a gap remains in effectively translating policies and recommendations into concrete actions, and practical and accessible tools in many national contexts, with various initiatives remaining fragmented or temporary.

**Figure 1:**
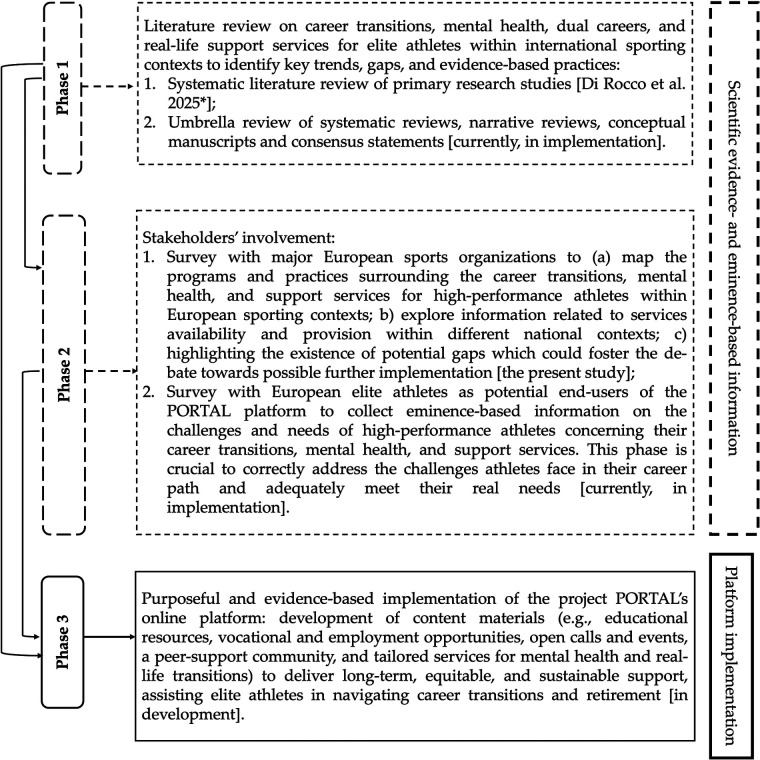
The project PORTAL's research and platform implementation design. *Manuscript published in Sports (60).

Digital innovation may help overcoming these barriers. In fact, online platforms, mobile applications, digital educational resources and counseling services might represent a scalable and cost-effective strategy to deliver support across borders and competitive contexts to a wider elite athletes’ population (57). These tools can integrate mental health screening, career guidance and mentoring, financial and health literacy, dual career counselling, and peer support, and address the complex and interconnected challenges of career termination (58). At the European level, policy initiatives and funding mechanisms are emphasizing the role of digital technologies in promoting accessibility, inclusivity, and lifelong support (59). With the goal of developing an inclusive online platform for elite athletes to effectively manage their retirement phase, the “Supporting Olympians Transitioning to Real Life in Concern of their Mental Health” (PORTAL) project has been recently financed by the European Commission within its Erasmus + Sport programme. The platform will be also complemented by a network of Real-Life Transition Officers, to deliver holistic support to elite athletes, with a particular focus on athletes facing fewer opportunities, providing a sustainable, long-term resource for retiring elite athletes. To succeed, this initiative requires the integration of robust, evidence-based knowledge drawn from both scientific literature (60) and best practices. In particular, to strengthen the foundation for sustainable approaches to elite athletes’ support and to substantiate the development of content materials (e.g., educational resources, vocational and employment opportunities, open calls and events, a peer-support community, and tailored services for mental health and real-life transitions) to be delivered though a digital platform transcending national boundaries and policy frameworks, there is a need of mapping key trends and identifying knowledge gaps and good practices. Hence, the project PORTAL online platform is meant to be grounded on a multidimensional and multi-sectorial body of information, including scientific evidence, best practices in place, and the elite athletes’ perceptions and opinions, as summarized in Figure 1. This approach would contribute to building long-term, equitable, and sustainable systems to assist elite athletes in navigating one of the most significant transitions of their lives. To provide a comprehensive picture of the challenges and needs high-performance athletes face in relation to their career transitions, mental health, and support services within different sporting and cultural settings, national information at European level is crucial. Therefore, the present study aimed at: a) mapping the programs and practices surrounding the career transitions, mental health, and support services for high-performance athletes within European sporting contexts; b) exploring information related to services availability and provision within different national contexts; c) highlighting the existence of potential gaps which could foster the debate towards possible further implementation within the project PORTAL platform.

## 2 Materials and methods

### 2.1 Study design and procedures

The present study was performed under the “Supporting Olympians Transitioning to Real Life in Concern of their Mental Health” (PORTAL) Project, financed by the ERASMUS + Sport programme (no. 101184857). The study aimed to effectively map the policies, programs, and practices surrounding high-performance athletes’ career services (e.g., career transitions, mental health, real-life support services, regulatory frameworks, services availability and provision, and challenges) within different European national contexts. Thus, a comprehensive (e.g., including several thematic areas related to the different career services) semi-structured electronic survey administration to major sports organizations was considered the most suitable methodology for the present study. In particular, electronic surveys are widely applied for geographically dispersed institutional respondents, while semi-structured formats (e.g., combining structured questions for quantitative data and open-ended questions for qualitative insights) allow balancing standardization with the flexibility needed to capture organizational complexity. In fact, electronic surveys present the following characteristics (61):

- practical and effective for gathering detailed and diverse information from different organizations;
- time and cost efficient, enabling faster data collection and processing (e.g., a crucial aspect for studies requiring timely results such those connected to funded projects with a short project lifetime);
- standard format, ensuring consistent question presentation and data collection across all respondents, reducing interviewer bias;
- easy to distribute and manage (e.g., reducing logistical challenges and allowing respondents to complete them at their convenience);
- adaptable (e.g., tailored to the specific needs of the study);
- anonymity and confidentiality features (e.g., in the context of major European sports organizations internal policies and practices, respondents may feel more comfortable providing honest answers in an electronic format, especially when sensitive or organizational data is involved).

The present study was conducted as a desk research or secondary research, encompassing a structured approach to gathering, analyzing, and synthesizing pre-existing information from various sources. This methodological approach has been selected to: i) fill-in the knowledge gap in relation to major practices in place across Europe to sustain elite athletes’ mental health, career transitions and career retirement; ii) to collect relevant information through a rigorous methodological approach; iii) to provide a scientific background to develop appropriate strategies and actions towards a sustainable impact in the field. In particular, for the present study, the key objectives were:

- to collect and review information across EU countries on mental health, career transitions, career retirement, and other support services to provide an evidence foundation for subsequent tasks and actions;
- to operate a cross-national comparison of strategies, policies, and performance in the area of elite athletes’ career assistance and support towards the identification of best practices to be exploited;
- to identify issues, challenges and gaps in elite athletes’ career assistance and support services within and among national contexts to be addressed through concrete, sustainable strategies and actions, including the project PORTAL's online platform implementation.

#### 2.1.1 Development process of the tailored survey for the present study

To meet research and practical implementation objectives, the following developmental stages were applied: 1) preliminary development; 2) refinement; 3) agreement and final version.

The first stage encompassed the preliminary definition of the tailored electronic survey, performed involving all project partners from five European countries (Croatia, France, Italy, North Macedonia, Romania). In particular, the instrument has been designed and structured coherently with research objectives and the specificity of respondents target group (e.g., major EU sports organizations). Since desk research depends on secondary data, verifying the credibility of sources is essential to ensure data accuracy. Thus, the survey structure has been conceived to include both closed-ended and open-ended items, complemented by adequate space for proper citation of sources and references to substantiate responses. To perform this task, an online focus group has been considered suitable to brainstorm ideas between partners representing different national contexts and organizational/professional profiles (e.g., sports organizations, academic institutions, mental health support). Thus, a preliminary version of the survey has been drafted including a pool of 22 items to collect the following information:

1. Organization's demographics (e.g., name, country, type, size, respondent's role within the organization, main activities performed in the field of career transitions, mental health, and support services for high-performance athletes);
2. Specific thematic sections concerning: i) mental health support services; ii) career transitions and retirement support services; iii) other available support services, if any; and iv) organizational challenges and opportunities in addressing high-performance athletes’ needs.

Each section has been designed to explore specific challenges and needs athletes may face, service provisions, formal regulations, subjective evaluation of service effectiveness, and any gaps that should be addressed.

The second stage was designed to refine the preliminary version of the tool, including all project partners. Revisions encompassed the final definition of the tool structure (e.g., number of sections, number of items), response typology (closed-ended; open-ended), items’ content concerning career transitions, mental health, and support services for high-performance athletes, and clarity of wording. Distance communication strategies (e.g., online meetings, email) have been used for the review process.

Lastly, a consortium agreement was applied to define the final version of the survey. This final phase encompassed the anonymous electronic rating of each section and item (e.g., 1–10 Likert-type scale) regarding its relevance, adequateness, and appropriateness in relation to the general and specific scopes of the study. Furthermore, space for open comments has been also provided to allow project partner respondents to propose revisions and changes to the items’ structure and content.

General results of the rating of the instrument suitability for the present study are presented in Table 1 and show a high degree of both perceived relevance and appropriateness of each survey section (range: 9.0–9.7 pts). Furthermore, collected requested revisions allowed to make changes to the tool structure by adding and/or changing items, enhancing the overall quality of the data collection. At the end of this process, the final English version of the survey has been reached, including a total of 34 items, as presented in the Supplementary Material 1.

**Table 1:** Scores of the consortium agreement rating task for the survey final definition.

Survey section	Score (pts.)	
	Mean	SD
Section 1 - General information		
Appropriateness	9.7	0.8
Section 2 - Mental Health Support Services		
Relevance (Gran Mean ± SD)	9.4	1.3
Appropriateness	9.2	1.6
Section 3 - Career Transitions Support Services		
Relevance (Gran Mean ± SD)	9.5	1.8
Appropriateness	9.8	0.4
Section 4 - Other Support Services		
Relevance (Gran Mean ± SD)	9.1	2.1
Appropriateness	9.0	2.4
Section 5 – Challenges and Opportunities		
Relevance (Gran Mean ± SD)	9.1	1.6
Appropriateness	9.5	0.8

#### 2.1.2 Administration procedures

The survey was circulated among European National Olympic Committees (NOCs). In particular, organization's specific departments and/or personnel involved in the provision of career transitions, mental health, and support services to high-performance athletes have been targeted as potential respondents. Potential respondents within European NOCs have been identified through project partners’ own networks and contacted electronically. Potential respondents were assured that there were no right or wrong answers to the proposed questions, that responses were kept confidential, that they could withdraw from the survey at any time and for any reason, and were asked to provide an informed consent to participate in the study. Each participant anonymously completed the survey, and the submitted responses were electronically archived and subsequently used for data analysis.

### 2.2 Respondents

The survey was sent to a total of 27 European NOCs. A total of 17 organizations representing their respective countries responded to the survey (e.g., response rate 63%). In particular, 16 NOCs and one National Sports Institute (e.g., French High Performance Sport Institute, INSEP) filled out the tool. General information of respondents (e.g., country, type of organization, number of members and employees) is presented in Table 2. The geographical representation portrays different policy approaches and practices in place towards elite athletes’ dual career and career assistance (22, 55, 56), ensuring a representative and diverse overview of career services. Among NOCs, respondents’ organization's size differs substantially in both number of members (e.g., range 32–250 member organizations) and number of employees (e.g., range 8–250). To note, the INSEP of France reported 1060 and 300 as number of member coaches/athletes and number of employees, respectively.

**Table 2:** General information of respondents to the electronic survey.

Country	Type of organization	Number of members	Number of employees
Belgium	NOC	–	25
Bosnia and Herzegovina	NOC	43	8
Croatia	NOC	75	75
Czechia	NOC	81	–
France	French High Performance Sport Institute	1060^a^	300
Germany	NOC	90	250
Italy	NOC	250	165
Kosovo	NOC	53	15
Latvia	NOC	40	10
Malta	NOC	250	10
Moldova	NOC	94	13
Montenegro	NOC	32	8
North Macedonia	NOC	21	21
Portugal	NOC	104	28
Romania	NOC	54	117
Slovenia	NOC	193	35
Spain	NOC	121	35

^a^ Athletes and coaches; - missing information.

### 2.3 Data analysis

National context, key information and insights, and major trends in relation to the main research themes were used to systematically organize collected data. Collected responses were grouped according to data type (closed-ended vs. open-ended items). Closed-ended items (e.g., multiple-choice, dichotomous) were analyzed quantitatively through frequencies of occurrence (n; %). Open-ended responses were examined qualitatively following six phases: familiarization, coding, theme development, refinement, naming, and reporting (62, 63). Raw comments were coded and organized into sub-themes. To reduce potential bias, two authors independently reviewed the material through multiple readings and discussed emerging themes until consensus was reached. In cases of disagreement, a third author was consulted (63). To generate a comprehensive picture of the state of the art of high-performance athletes’ career transitions, mental health, support services management, and good practices across countries, the main findings are reported in relation to individual national contexts and collective (European) trends. In particular, results were organized to provide: i) collective information in relation to gaps and challenges athletes face in their career path, with a specific focus on transition out of elite sport; and ii) a descriptive overview of similarities and/or discrepancies between countries.

## 3 Results

An organization's is a critical aspect in determining the operativity, capacity, and availability of services to address top-performance athletes career needs and assistance, determining a variety of practices and approaches in place within different national contexts. Preliminarily, respondents were asked to declare whether their organization is active in supporting top-performance athletes through different career assistance services. Results are presented as individual national responses in Table 3, which shows that the majority of respondents reported ongoing or in development efforts and services to assist their top-performance athletes. Among respondent countries, Italy, Latvia and Malta reported several problems in providing assistance services to their athletes. Although these findings preliminarily provided a positive image in relation to the proactive organizational involvement in supporting elite athletes during and after their sport career, responses to the thematic survey sub-sections (e.g., mental health services, career transitions services, additional support services) revealed several issues and obstacles in ensuring adequate support in many countries.

**Table 3:** Individual responses of services provision to top-performance athletes.

Country	Career transitions services	Career retirement services	Mental health support services	Additional support services
Belgium	Yes	Yes	Yes	Yes
Bosnia and Herzegovina	In development	In development	No	Yes
Croatia	Yes	Yes	Yes	Yes
Czechia	Yes	Yes	Yes	Yes
France	Yes	Yes	Yes	Yes
Germany	In development	Yes	Yes	Yes
Italy	Yes	No	No	Yes
Kosovo	In development	No	Yes	Yes
Latvia	No	No	Yes	No
Malta	No	No	In development	In development
Moldova	In development	In development	No	No
Montenegro	In development	In development	Yes	Yes
North Macedonia	Yes	Yes	Yes	Yes
Portugal	Yes	Yes	Yes	Yes
Romania	In development	Yes	In development	Yes
Slovenia	Yes	Yes	No	Yes
Spain	Yes	Yes	Yes	Yes

### 3.1 Mental health support services

Regarding mental health support services provision, Tables 4, 5 show an encouraging trend towards the integration of mental health support within elite sport systems across respondent European countries. All countries except Bosnia and Herzegovina, Italy, and Moldova reported mental health services, despite the nature and scope of this provision vary widely. Whilst France, Portugal, Romania, and Spain offer comprehensive services that include in-house professionals, peer support, awareness training, and crisis intervention, reflecting mature mental health systems, Czechia, Malta, and North Macedonia, rely predominantly on access to external professionals.

**Table 4:** Mental health service provision and typology for each respondent country.

Country	Service provision	Typology	Additional information (open comment)
Belgium	Yes	▪ In-house sports psychologists or counsellors ▪ Access to external mental health professionals ▪ Crisis intervention services	Direct support during the Olympic Games, indirect support by facilitating athletes during the out-of-delegation period
Croatia	Yes	▪ Access to external mental health professionals ▪ Peer support programmes ▪ Crisis intervention services	Aside from the regular mental health support that the Croatian Olympic Committee provides for its top-performing athletes, a sports psychologist is a mandatory member of every delegation at the Olympic Games and other major sporting events
Czechia	Yes	▪ Access to external mental health professionals	We cooperate with mental coaches as well as sports psychologists. At the Olympics 2024 we had accredited a welfare officer on site
France	Yes	▪ In-house sports psychologists or counsellors ▪ Access to external mental health professionals ▪ Peer support programmes ▪ Mental health awareness training (and education) for athletes, coaches and staff ▪ Crisis intervention services ▪ Research	The French law demands a mandatory Psychology surveillance for all athletes at least once a year
Germany	Yes	▪ Access to external mental health professionals	NA
Kosovo	Yes	▪ Mental health awareness training for athletes, coaches and staff	NA
Latvia	Yes	▪ In-house sports psychologists or counsellors	Available sports psychologist consultations
Malta	Yes	▪ Access to external mental health professionals	NA
Montenegro	Yes	▪ Access to external mental health professionals	Although no in-house sports psychologist is employed, the MOC provides access to external mental health professionals upon request, particularly for athletes competing at the highest levels (national team athletes). Additionally, we will introduce mental health awareness training as part of educational programs for athletes, coaches, and support staff, ensuring a proactive approach to mental well-being. Our long-term objective is to integrate mental health support more systematically by collaborating with sports federations and experts to provide tailored psychological services for all elite athletes
North Macedonia	Yes	▪ Access to external mental health professionals ▪ Mental health awareness training for athletes, coaches and staff	NA
Portugal	Yes	▪ In-house sports psychologists or counsellors ▪ Access to external mental health professionals ▪ Peer support programmes ▪ Mental health awareness training for athletes, coaches and staff	NA
Romania	Yes	▪ In-house sports psychologists or counsellors ▪ Access to external mental health professionals ▪ Peer support programmes ▪ Mental health awareness training for athletes, coaches and staff ▪ Crisis intervention services	NA
Slovenia	Yes	▪ Access to external mental health professionals ▪ Mental health awareness training for athletes, coaches and staff	We organize sports psychology workshops for young athletes preparing to compete in multi-sport competitions. We also have a list of so-called “reference experts” who provide psychological support to athletes (sports psychologists)
Spain	Yes	▪ In-house sports psychologists or counsellors ▪ Access to external mental health professionals ▪ Peer support programmes ▪ Mental health awareness training for athletes, coaches and staff	NA
Bosnia and Herzegovina	No		
Italy	No		
Moldova	No		

NA, not applicable.

**Table 5:** Collective overview of provided mental health services.

Typology	Frequency of occurrence	
	(*n*)	(%)
Access to external mental health professionals	12	34.3
Mental health awareness training (and education) for athletes, coaches and staff	7	20.0
In-house sports psychologists or counsellors	6	17.1
Peer support programmes	5	14.3
Crisis intervention services	4	11.4
Other (Research)	1	2.9

Total collected citations *n* = 35.

Furthermore, the synthesis presented in Table 5 highlights that the most common service across countries is access to external mental health professionals (34.3%), followed by mental health awareness training (20.0%) and in-house psychologists (17.1%), whereas underdeveloped community-based and reactive support mechanisms are reflected in the relatively low prevalence of crisis intervention (11.4%) and peer support (14.3%).

Regarding major perceived mental health challenges faced by top-performance athletes especially during the retirement period (total collected citations *n* = 35), anxiety, loss of identity, and depression were reported by 15.6% of respondents. Additional concerns included difficulties in adjusting to a new routine (9.4%) and career uncertainty (6.3%), highlighting the psychological burden athletes face during the retirement transition.

Moderate awareness levels (score: 6.7 ± 2.2 pts) emerged regarding the respondents’ perception of high-performance athletes’ awareness of their mental health support needs. However, the majority of organizations (47%) do not consider evaluation of mental health needs, 24% are still developing monitoring tools, and only 29% performed formal assessments, undermining the ability to deliver targeted, individualized care and to track emerging mental health issues within the elite sportspersons. Furthermore, a fragmented picture emerged in relation to assessment methodologies (Table 6). France and Romania demonstrated best practices through multi-method approaches, including anonymous surveys, regular check-ins, and self-referral options. In contrast, other countries (e.g., Bosnia and Herzegovina, Italy, Malta) reported no assessment practices in place, reflecting a tendency towards informal or reactive strategies, with the most commonly cited assessment methods encompassing regular check-ins with coaching staff (20%), in-dividual counselling sessions (16%), and self-referral processes (16%). Furthermore, 32% of responses indicated no existing assessment methods, suggesting an urgent need to build standardized, proactive screening frameworks across national systems. Few respondents provided also further information, as reported in the following quotes:

**Table 6:** Reported methods for mental health needs assessment.

Country	How mental health needs are assessed:
Belgium	▪ Regular check-ins with coaching staff
Bosnia and Herzegovina	*
Croatia	▪ Regular check-ins with coaching staff ▪ Individual counselling sessions
Czechia	*
France	▪ Regular check-ins with coaching staff ▪ Anonymous surveys or assessments ▪ Individual counselling sessions ▪ Athlete self-referral process
Germany	*
Italy	*
Kosovo	▪ Anonymous surveys or assessments
Montenegro	*
Latvia	*
Malta	*
Moldova	*
North Macedonia	▪ Individual counselling sessions ▪ Athlete self-referral process
Portugal	▪ Athlete self-referral process ▪ Regular contact with athletes
Romania	▪ Regular check-ins with coaching staff ▪ Anonymous surveys or assessments ▪ Individual counselling sessions
Slovenia	*
Spain	▪ Regular check-ins with coaching staff ▪ Athlete self-referral process

* No assessment practices are in place.

**Croatia:** The mental health needs of top-performing athletes are an important focus for our organization. While we provide general support and recognize the importance of psychological well-being, we are continuously working on improving our assessment methods and developing more structured approaches to better address their specific needs.

**Portugal:** Through the Athletes’ Office, regular contact is maintained with athletes in Olympic preparation or Olympic athletes, identifying their needs and connecting them with support structures to address those needs. The NOC has a coordinator for psychological services responsible for overseeing the provision of support solutions and training in this area.

Funding sources to sustain athletes’ mental health support services were evenly split between public funds (35%) and own organizational funds (35%), with some countries utilizing Olympic Solidarity funds or private sponsorship. In considering that solid funding sources is crucial to ensure and sustain accessible, quality, and long-term and services, a lack of funding (41.2%) emerged as the primary barrier to provide mental health services to top-performance athletes. Stigma, lack of available ser-vices, and lack of professionals each accounted for 17.6% of responses, reflecting structural and cultural challenges and suggesting that awareness exists but is not yet fully matched by capacity or resourcing. When services are available, they are offered to both Individual and Team sports athletes (71%) and are perceived as somewhat (47%) and very (35%) effective. Service provision is largely targeted at medalist athletes and top-performance athletes competing in major continental and world sport events, potentially excluding young-er or lower-tier elite athletes who may also be at risk of mental health issues. Despite France and Slovenia consider also youth athletes, the widespread primarily focus on the most decorated athletes may represent a bias towards equitable provision and access to mental health support. Thus, this approach grants performance outcomes rather than sustaining the path towards excellence in elite athletes, which might require several years. Further comments have been provided by few respondents:

**Croatia:** The biggest obstacle to accessing mental health services for athletes in our organization is the lingering stigma around seeking psychological support, as many athletes still hesitate to reach out due to fear of being perceived as weak or vulnerable. While we have mental health professionals available, another challenge is budget limitations—although support exists, a larger budget would allow for a more comprehensive approach, making it easier to provide continuous psychological care and expand the availability of specialized experts.

**Czechia:** Czech NOC has also special division for Dual career of athletes where they can request a support for further their education to be able to transition better into their after-sport career.

**France:** INSEP develops this topic and capacity building thank to ERASMUS program (e.g.: MENTIS, EUPHORIA, PORTAL, etc.)

### 3.2 Career transition services

Regarding career transitions support services provision, results showed that only 35% of countries currently provide career transition services, whereas 35% declaring to be in a developmental phase, and 30% without any services, indicating an in-consistent implementation of this crucial service. When provided, the most common types of service are educational workshops (29%), followed by career counselling (19.4%), and competence/skills development programs (16.1%). Services such as financial planning, job placement, and networking opportunities were less frequently reported (9.7% each), de-spite being crucial for reducing career uncertainty after elite sport retirement. The low presence of monitoring tools (3.2%) signals a lack of structured follow-up mechanisms. A deeper look into individual respondents’ answers (Table 7) showed that only few countries (e.g., France, Spain, Portugal, and Czechia) offer multi-faceted programs combining several types of services. However, seven countries reported no or underdeveloped services, showing significant disparity in services provision across Europe. Informal, *ad hoc* systems dominate in some nations (e.g., Romania, Croatia), whereas others (e.g., Slovenia and Belgium) have institutionalized partnerships and funding models, reflecting a stronger commitment and a structured approach. Several countries complemented their answers by providing further comments:

**Table 7:** Career transitions services provision and typology for each respondent country.

Country	Career transition services provision:	Typology
Belgium	Yes	▪ Networking opportunities with industry professionals ▪ Educational workshops (e.g., resume building, interview skills)
Bosnia and Herzegovina	In development	▪ Job placement services ▪ Educational workshops (e.g., resume building, interview skills)
Croatia	In development	
Czechia	Yes	▪ Career counselling services, Competences/Skills development programs ▪ Educational workshops (e.g., resume building, interview skills)
France	Yes	▪ Career counselling services ▪ Job placement services ▪ Competences/Skills development programs ▪ Competences/Skills assessment/monitoring tools ▪ Educational workshops (e.g., resume building, interview skills)
Germany	In development	▪ Career counselling services
Italy	No	
Kosovo	No	
Latvia	No	
Malta	No	
Moldova	No	▪ Educational workshops (e.g., resume building, interview skills)
Montenegro	In development	
North Macedonia	In development	▪ Competences/Skills development programs, ▪ Educational workshops (e.g., resume building, interview skills) ▪ Financial planning and management support
Portugal	Yes	▪ Career counselling services ▪ Competences/Skills development programs ▪ Educational workshops (e.g., resume building, interview skills) ▪ Financial planning and management support
Romania	In development	▪ Networking opportunities with industry professionals ▪ Other (Support in becoming a coach; International courses and master programs supported by the IOC; Partnerships with army and Romanian information services)
Slovenia	Yes	▪ Career counselling services ▪ Educational workshops (e.g., resume building, interview skills) ▪ Financial planning and management support
Spain	Yes	▪ Career counselling services ▪ Job placement services ▪ Competences/Skills development programs ▪ Networking opportunities with industry professionals ▪ Educational workshops (e.g., resume building, interview skills)

**Belgium:** Career transition support services are organized within the communities but it's our role to connect athletes with the existing initiatives. Every 4 year we organize in collaboration with the communities an Athlete Career Day focusing on (the preparation of) the transition out of sport. We also have the ACP Fund (funded by the Olympic Solidarity) were we use $10.000/4years to reimburse Olympians’ who choose to follow a course/education/training.

**Croatia:** Our organization is currently developing career transition support services for high-performance athletes moving from professional sports to other career paths. Once fully implemented, these services will be available to athletes with Olympic, World, and European achievements.

**Czechia:** Services are available to all high-performance athletes. In case of high demand, priority is given to Olympians and Olympic medalists.

**France:** In our organization, a specific department is dedicated to this job (career transitions services provision).

**Portugal:** Through the Athletes’ Office, training and awareness initiatives on career transition are developed. At the same time, close follow-up is provided to athletes to activate available support measures and existing programs.

**Romania:** Our approach is mostly informal and non-structured, provided in case of athlete's needs and if asked. Still no structured policy is developed.

**Slovenia:** We have an agreement with an institute that provides comprehensive support to athletes in the phase of ending their careers and transitioning to a post-sports career. As part of the Athletes’ Forums and Olympic Camps, we also organize various useful workshops (financial literacy, CV, etc.).

Post-retirement services provision and access vary widely among countries. In particular, France, Slovenia, and Portugal offer unlimited access, aligning with athlete-centered long-term support. In contrast, Italy, Malta, and Moldova offer no post-retirement services, and other countries (e.g., Czechia, Croatia) reported access “depending on individual needs” – a subjective and potentially inequitable criterion. Hence, the lack of standardized duration in many cases could affect continuity and quality of career assistance throughout an elite sport career transition. Additional information has been provided by several respondents to deepen their answers:

**Bosnia and Herzegovina:** It depends on the available programs at the given moment.

**Croatia:** Access to ongoing career transition support services after retirement from competitive sports depends on the individual athletes and their specific situation, as each case is quite unique. For example, here at the Croatian Olympic Committee, we have several former high-performance athletes employed, and through their work with the Croatian Olympic Committee, they have gained many opportunities to transition into new career paths.

**Czechia:** It depends on the type of support requested. Financial support of education can be requested until 2 years after retiring. Other services don't have time limitation yet.

**France:** An athlete may be registered in the “Reconversion” category if they have been listed as a high-level athlete in the Elite category or have been on this list for four years, with at least three years in the Senior category, and if they no longer meet the conditions for registration in the Elite, Senior, or Youth categories while presenting a professional integration project. Registration in the “Reconversion” category is valid for one year and may be renewed for the same duration, up to a maximum of five years.

**Portugal:** The focus of action is to respond to the individual needs of each athlete, regardless of the stage of their career or life.

**Romania:** 17 for informal support.

**Slovenia:** If athletes express interest, we include them in the programs implemented by the institute.

Regarding high performance athletes’ awareness of their career transition needs, a perceived moderate awareness emerged (score: 5.5 ± 2.6 pts), suggesting a perceived knowledge gap that could delay proactive planning. When services are available, their perceived effectiveness range from “very effective” (Belgium, Kosovo, Latvia) to “not effective at all” (Italy, Romania), revealing striking contrasts. While most countries serve both individual and team sport athletes, availability and perceived effectiveness might remain disconnected, indicating room for evidence-based evaluation and quality improvement (Table 7). Further additional insights have been provided by a few respondents:

**Croatia:** Career transition support services are crucial for high-performance athletes, as they provide the necessary resources and guidance to successfully navigate the shift from sports to other career paths. Many of our athletes possess significant “business” potential that has been suppressed due to years of dedication to their sports. After their sporting careers, it's essential to help them reconnect with this untapped potential, allowing them to pursue fulfilling careers and make a successful transition. Such support would enable athletes to leverage their discipline, teamwork, and leadership skills in new professional environments, ultimately helping them thrive in post-sport life.

**Portugal:** Athletes are, to some extent, aware of the importance of timely preparation for career transition. A large percentage of athletes develop dual career plans during their sporting careers. One of the main current challenges is reducing the gap between the completion of academic studies and integration into the job market, while maintaining a connection to their field of study through participation in initiatives, networking opportunities, internships, shadowing experiences, and similar activities.

### 3.3 Additional support services

Table 8 reports the additional support services provision. The most frequently offered ser-vices resulted nutrition counselling (21.4%), financial aid and budgeting support (19.0%), social support (19.0%), and travel/logistics support (19.0%), which reflect practical needs closely linked to athletes’ performance and well-being. Services like legal assistance (9.5%), family support (7.1%), and housing (2.4%) resulted less frequent, despite their importance during transitional or crisis periods. The dominant access pathways resulted the internal organizational support networks (40%) and coach/staff referrals (33.3%), with fewer ser-vices accessible via self-referral (20%). This suggests that athletes’ access is often dependent on the initiative of staff or the strength of the institutional infrastructure, which may pose a barrier for athletes uncomfortable seeking help or unfamiliar with internal processes. Inclusion of mechanisms such as Athlete Commissions or National Federations (6.6%) reflects limited but important alternative pathways. A deeper look into individual respondents’ answers showed that a few countries only (e.g., France, Spain, and Croatia) offer a broad range of services (e.g., financial, legal, nutrition, family, and social support), demonstrating comprehensive, athlete-centered systems. Some countries (e.g., Malta, Kosovo) reported limited or unspecified services, pointing to inconsistency in provision across Europe. Targeted recipients are mainly medalists or top-performance athletes, though countries like Slovenia and France having a more inclusive approach for youth athletes. Finally, Belgium, Croatia, Portugal, and France provide multi-channel access including internal networks, coach referrals, and self-referrals, offering flexible pathways for support provision. A few countries provided also further comments to deepen their answers:

**Table 8:** Additional support services provision for each respondent country.

Country	Additional support services	Target recipients	Access modality
Belgium	▪ Financial aid and budgeting support ▪ Nutrition counselling	▪ Medalists in major international competitions (European, World, Olympic) ▪ Olympians	▪ Directly through the organization's internal support network ▪ Via referrals from coaches or staff ▪ Self-referral process
Bosnia and Herzegovina	▪ Travel and logistics support ▪ Other (scholarships)	▪ Medalists in major international competitions (European, World, Olympic) ▪ Elite athletes competing at the highest continental and world level	▪ Through National Federations and by their own initiative
Croatia	▪ Financial aid and budgeting support ▪ Travel and logistics support ▪ Social support services (e.g., community building, social networks)	▪ Medalists in major international competitions (European, World, Olympic)	▪ Directly through the organization's internal support network ▪ Via referrals from coaches or staff ▪ Self-referral process
Czechia	▪ Nutrition counselling ▪ Social support services (e.g., community building, social networks)	▪ It depends. Social networking events are available to all participants of World and Olympic/Paralympic games. ▪ Nutrition is available for the TOP TEAM members	▪ Self-referral process
France	▪ Housing assistance ▪ Financial aid and budgeting support ▪ Legal or contractual assistance ▪ Family support services ▪ Nutrition counselling ▪ Social support services (e.g., community building, social networks)	▪ Medalists in major international competitions (European, World, Olympic) ▪ Younger athletes	▪ Directly through the organization's internal support network ▪ Via referrals from coaches or staff ▪ Self-referral process
Germany	▪ Financial aid and budgeting support ▪ Legal or contractual assistance ▪ Nutrition counselling ▪ Travel and logistics support	▪ Medalists in major international competitions (European, World, Olympic)	▪ Directly through the organization's internal support network ▪ Via referrals from coaches or staff
Italy	▪ Travel and logistics support		▪ Via referrals from coaches or staff
Kosovo		▪ Medalists in major international competitions (European, World, Olympic) ▪ Elite athletes competing at the highest continental and world level	▪ Directly through the organization's internal support network ▪ Via referrals from coaches or staff
Latvia	▪ Nutrition counselling ▪ Travel and logistics support ▪ Social support services (e.g., community building, social networks)		▪ Directly through the organization's internal support network
Malta			
Moldova	▪ Social support services (e.g., community building, social networks)	▪ Medalists in major international competitions (European, World, Olympic)	▪ Via referrals from coaches or staff
Montenegro	▪ Financial aid and budgeting support ▪ Nutrition counselling ▪ Travel and logistics support	▪ Medalists in major international competitions (European, World, Olympic) ▪ Elite athletes competing at the highest continental and world level	▪ Directly through the organization's internal support network ▪ Via referrals from coaches or staff
North Macedonia	▪ Financial aid and budgeting support	▪ Medalists in major international competitions (European, World, Olympic)	▪ Directly through the organization's internal support network
Portugal	▪ Legal or contractual assistance ▪ Family support services ▪ Nutrition counselling ▪ Social support services (e.g., community building, social networks)	▪ Medalists in major international competitions (European, World, Olympic) ▪ Elite athletes competing at the highest continental and world level	▪ Directly through the organization's internal support network ▪ Via referrals from coaches or staff ▪ Self-referral process ▪ Other (Through the Athletes Commission)
Romania	▪ Nutrition counselling ▪ Social support services (e.g., community building, social networks)	▪ Medalists in major international competitions (European, World, Olympic) ▪ Junior athletes from NOCs Olympic centers	▪ Directly through the organization's internal support network ▪ Via referrals from coaches or staff ▪ Self-referral process
Slovenia	▪ Financial aid and budgeting support ▪ Travel and logistics support	▪ All interested, with priority given to medalists from major competitions ▪ Youth athletes preparing for multi-sport events	▪ Directly through the organization's internal support network
Spain	▪ Financial aid and budgeting support ▪ Legal or contractual assistance ▪ Family support services ▪ Nutrition counselling ▪ Travel and logistics support ▪ Social support services (e.g., community building, social networks)	▪ Elite Spanish and refugee athletes in Spain	▪ Directly through the organization's internal support network

**Croatia:** High-performance athletes in our organization receive support through various services. They benefit from categorization, which provides financial support based on their sporting achievements. We also have a professional nutritionist at the Croatian Olympic Committee who is almost always available for nutrition counselling. Additionally, we handle logistics and travel arrangements for major competitions such as the Olympic Games. Athletes also have access to social support through networking opportunities at events organized by the Croatian Olympic Committee. These services are designed to support athletes in both their athletic and personal development.

**Latvia:** A special organization has been established to address elite athletes’ needs and necessary support.

**Portugal:** The focus of action is the search for individualized solutions tailored to the specific needs of each athlete.

Athletes were perceived as moderately aware (score: 6.6 ± 1.4 pts) regarding their support services needs. Furthermore, when services are available, they are offered to both Individual and Team sports athletes (69%) and are perceived as somewhat (50%) and very (31%) effective. However, results suggest the growing but uneven landscape of additional support services for elite athletes across European responding countries. Whilst some countries offer diverse, structured, and accessible support systems, others lack even basic provisions. Access mechanisms are generally embedded within the organizations, which is efficient but may hinder autonomy and equal access if athletes are not fully informed or empowered. Efforts to standardize minimal service offerings, increase visibility, and encourage self-referral pathways could help address these gaps.

### 3.4 Challenges and opportunities in relation to athletes’ support services

Regarding major challenges and opportunities towards implementation of services, results showed that 63% of the respondent organizations have no regulatory framework explicitly defining career assistance practices (e.g., Bosnia and Herzegovina, Croatia, Czechia, Italy, Kosovo, Latvia, Malta, Moldova, Romania). Among them, Croatia, Kosovo, Romania, and Slovenia only reported temporary initiatives/activities. This aspect suggests that career assistance practices often rely on temporary initiatives or informal support. The absence of a structured approach impedes consistency and sustainability in service provision in the long term, as evidenced by Croatia's and Slovenia's acknowledgment of the need for improvement despite their informal efforts. In this regard, Croatia integrated with the following comment:

“At our organization, there is no specific regulatory framework explicitly defining career assistance practices for top-performance athletes. However, despite this, we strive to provide athletes with opportunities and support to help them continue developing after their sports careers. We are committed to ensuring that they have access to the resources and guidance they need to successfully transition into new career paths and build fulfilling lives beyond sport.”

Conversely, Belgium, France, Germany, Montenegro, North Macedonia, Portugal, and Spain reported a formal and structured approach to top-performance athletes’ career assistance.

Regarding services dissemination and athlete reach of available support measures, a fragmented picture emerged, suggesting disparity between promotion efforts and actual athlete reach. Whilst many Croatia, France, and Portugal actively promote their services, the proportion of athletes reached remains low, with most of the countries reporting less than 50% engagement. Only Croatia reported services reaching at least 75% of the eligible athletes, suggesting an effective communication strategy. Other countries, such as Italy and Czechia, showed low awareness accompanying the lack of services provision, highlighting the critical need for more robust approaches to athletes’ care and outreach mechanisms. Moreover, the disconnect between service promotion and athletes reach likely stems from inconsistent communication, limited program visibility, and perhaps low perceived value among athletes. In this regard, a few countries provided additional comments to deepen their answers:

**Croatia:** We always strive to provide the best possible support for our athletes, ensuring that their needs are met in every aspect of their athletic journey. However, we recognize the importance of continuously improving the services we offer. Our organization is actively working on enhancing these services to better support our athletes and help them succeed both in their sports and in their personal lives. We are committed to making ongoing improvements to ensure our athletes have the resources they need to excel. Almost all of our high-performance athletes utilize the services we provide, and we are confident that nearly all of them are aware of these services. However, we believe it is always important to ensure that the full range of support available to athletes is actively communicated. To that end, we make it a priority to publish and share information about the services offered by the Croatian Olympic Committee, so that athletes are fully informed and can take full advantage of what is available to them. Although we don't face any significant obstacles in supporting high-performance athletes, one of the ongoing challenges is ensuring that all athletes are fully aware of the wide range of services and support available to them. While we strive to provide comprehensive support, it is always beneficial to continue expanding and improving how we share information about these resources

**Czechia:** Different projects have different target population and there are no available data on this. My suggestion would be around above 50% for the TOP TEAM members and less than 25% for other projects.

Regarding the main organizational challenges in providing support to top-performance athletes (total collected citations *n* = 21), the lack of financial resources (38.1%), the lack of opportunities to connect with athletes and share information (23.8%), the lack of human resources to be involved in athletes’ career assistance (14.3%), and the lack of a holistic approach to athletes’ career management (9.5%) resulted the most critical aspects to be addressed. In particular, the lack of financial resources has been highlighted as constant and persistent issue in implementing athlete services, complemented by gaps in communication strategies to enhance information sharing and athlete engagement, determining challenges in outreach and visibility of available services. Furthermore, human resource constraints also limit organizations’ ability to deliver and maintain services. These findings suggest that even when good intentions exist, execution and/or implementation might fail due to systemic and/or structural deficiencies.

Regarding the perceived necessary organizational improvements or additional support services that would most benefit top-performance athletes (Table 9), recommendations encompass both service enhancement and communication improvement. In particular, Czechia, Croatia, and France advocate for better information provision about existing services, reflecting ongoing gaps in visibility. Other countries (e.g., Italy, Latvia, Montenegro, Slovenia) emphasize the need for the implementation of new services, including mental health, dual career, career transition, and peer mentorship programs, especially in consideration of the current absence and/or limited development of these services. Another interesting emerging trend is the need for shifting from basic provision to a more holistic and integrated support ecosystem, including services tailored to post-career and well-being aspects. Notably, Spain suggests deeper engagement of companies, highlighting the importance of external stakeholders in bridging the transition between sport and the workforce. To solve these challenges, recommended inter-institutional partnerships and collaborations call for a multi-sectoral approach, reflecting a broad recognition that effective athlete support requires inter-institutional collaboration. This should include the educational sector (e.g., Spain, Slovenia, Italy) targeting dual career programs planning and implementation. Also, collaborations with mental health organizations (e.g., Croatia, Slovenia, Spain) emphasize the need to adequately address psychological well-being throughout elite sport careers. Other interesting inputs include companies (Montenegro) and career development agencies (Croatia), suggesting forward-thinking efforts to use technology and career services to support athletes’ transitions. Finally, countries like France and Czechia acknowledge the need to strengthen coordination within their own NOC structures, suggesting internal fragmentation can also be a limiting factor.

**Table 9:** Recommended implementation and partnerships/collaborations foreseen as beneficial to implement athletes support services for each respondent country.

Country	Recommended implementation	Suggested partnerships or collaborations
Bosnia and Herzegovina	▪ Structured governmental support	▪ State institutions
Croatia	▪ Improving athletes’ information provision about their rights and the support available to them (particularly in terms of financial, social, and athletic assistance). Ensuring that athletes are fully aware of the resources and opportunities they have access to is crucial for maximizing their potential and well-being. Regular communication and clear information sharing about these services would help athletes make the most of the support offered to them by our organization.	▪ Educational institutions for dual career programs ▪ Mental health organizations for professional psychological support ▪ Career development agencies for post-retirement professional development and opportunities ▪ Health and wellness organizations for nutrition, rehabilitation, and injury prevention services
Czechia	▪ Dual career consultants ▪ Events where athletes can be in direct contact with NOC sponsors and other companies interested in hiring athletes ▪ Improved communication about the already available services ▪ Role of welfare officer during and after the Olympic Games	▪ Networking opportunities outside of sport
France	▪ A better communication about services availability	▪ Synergies with French NOC and NPC
Italy	▪ Services provision (Post career and mental health services for high level athletes)	▪ Educational bodies ▪ Sport ministry ▪ Private bodies
Kosovo	▪ Qualified sports psychologist within the organization	▪ Health institutions (department of Psychology) ▪ Educational bodies for dual career programs
Latvia	▪ Services provision (Dual career, career transition, mental health, nutrition etc.)	
Montenegro	▪ Services provision (dual career support, career transition support, financial support.)	▪ National Institute for sport medicine ▪ Companies providing user friendly tools for the athletes ▪ Private medical centers
Slovenia	▪ Services provision (peer mentorship networks, training to acquire certain competencies, workshops, financial support for dual career etc.)	▪ Employment agencies ▪ Mental health organizations
Spain	▪ Support form companies	▪ Educational bodies ▪ Mental health organizations ▪ Companies

## 4 Discussion

The present study provides an overview of how European National Olympic Committees (NOCs) and related organizations address the critical issues of mental health, career transitions, and broader support services for elite athletes. Findings revealed both encouraging developments and persistent gaps, reflecting a fragmented and uneven landscape across countries.

Across all surveyed themes, mental health support services are among the most widely acknowledged but inconsistently implemented. Although the majority of countries offer at least one form of mental health support, only few countries provide structured, multi-faceted programs encompassing awareness training, in-house professionals, peer support, and crisis intervention. Assessment practices remain underdeveloped in many countries, with 47% reporting no formal evaluation process and 32% lacking any assessment tools. In particular, a key finding is the variability in the provision of mental health services, confirming previous literature in this field (55). Whilst most countries report some form of provision, services are often limited to access to external professionals, with crisis intervention and peer support mechanisms underdeveloped. This result reinforces concerns raised by the IOC (27, 29, 30) that athlete mental health remains insufficiently integrated into organizational structures, often addressed reactively rather than proactively. The lack of systematic needs assessments in the majority of NOCs further undermines the possibility of delivering targeted and evidence-based interventions. These gaps also align with previous literature in this area, highlighting stigma, structural barriers, and limited resources as persistent obstacles to athlete mental health care (47, 64). Furthermore, the absence of standardized diagnostic frameworks and proactive monitoring might lead to neglecting emerging vulnerabilities, particularly during sensitive phases such as career termination (20, 24).

In terms of career transitions services, progress appeared slower and less uniform. In fact, only 35% of countries offer established programs, whereas several countries are still developing them. Provision typically includes educational workshops and counselling, but the absence of tools for long-term follow-up, financial planning, and job placement weakens the transition ecosystem. Moreover, few countries offer unlimited access to services after retirement, and the subjective “case-by-case” nature of access in many countries may impact services provision and accessibility. Hence, the results also confirm that career transition services remain underdeveloped, with only a minority of NOCs providing structured programs including more diversified career services. Even where services exist, follow-up mechanisms and duration of support are inconsistent, despite the extensive evidence that dual career pathways facilitating the combination of sport and academic careers and proactive preparation during active sporting years are among the most effective protective factors for post-retirement adaptation (18, 23). In fact, dual career athletes more likely report smoother transitions, stronger networks, and lower risks of mental health issues (44–46). This finding reaffirms a gap in translation of evidence into concrete actions. However, organizational awareness towards these issues is certainly growing, with many NOCs operating with *ad hoc* or informal systems when lacking financial resources and/or a structured strategy and policy framework for elite athletes’ support. However, temporary and fragmented initiatives may exclude large numbers of athletes, particularly those outside the most decorated groups (medallists in major competitions and Olympians) from adequate support (49, 65, 66). This performance-based bias raises ethical questions, as it reinforces inequalities and undermines the sustainability of support structures (55).

Additional support services, such as nutrition counselling, financial aid, and social support, were more frequently provided but still inconsistent across contexts. Services addressing legal, family, or housing needs were rare, despite their potential importance during transitional phases. This limited support approach characterizing the majority of respondent countries contrasts with the widespread call for holistic, multidimensional support frameworks that integrate mental, social, vocational, and practical assistance (18, 67). Importantly, many NOCs reported reliance on internal networks or coach referrals as main access routes, potentially creating barriers for athletes reluctant to seek help or unaware of their entitlements (47, 68–70). This highlights the need for transparent communication, self-referral options, and athlete empowerment to foster equitable access (27, 28, 30, 71).

Organizational challenges span several domains. Notably, 63% of countries lack a regulatory framework to structure their service offerings, relying instead on *ad hoc* or temporary initiatives. This might correlate with inconsistencies and long-term sustainability in service quality. Across all domains, financial constraints emerged as the most frequently cited barrier (38.1%) towards extensive and effective athletes’ support services implementation, alongside insufficient human resources and organizational fragmentation. Even when services exist, communication and visibility remain problematic, with most countries reaching less than 50% of their athlete populations. These findings confirm previous research findings stressing that the sustainability of athletes’ welfare systems depends on resource allocation, long-term funding, and institutional commitment (21, 50). Whilst some organizations reported leveraging Olympic Solidarity or ERASMUS + Sport funding, the reliance on temporary projects or sponsorship undermines continuity and scalability. Thus, embedding athlete support within stable national frameworks, aligned with international standards (6, 9, 10, 27, 30, 35, 36) remains a pressing challenge.

Results of the present study confirmed a persistent gap in services provision and reach, with both country-specific and collective features. For mental health support services, resulted the most widely acknowledged, inconsistent implementation and reactive approaches challenge effective and equitable support of elite athletes during their different career stages. For career transitions and additional support services, temporary and fragmented initiatives remain the most frequently applied. Finally, the frequent lack of a national and/or organizational regulatory framework to structure service offerings, financial constraints, insufficient human resources dedicated to elite athletes’ support and gaps in communication/visibility of available services reflect a fragmented and uneven landscape across the participating countries. These findings have important implications for good governance implementation in sport settings. As highlighted by the Council of Europe (1, 3), good governance in sport encompasses not only integrity and accountability but also the protection of athletes’ human rights, including decent work and occupational safety (53). The inconsistent implementation of career assistance frameworks observed in the present study still reflects a broader policy–practice gap, where international recommendations (18, 31, 72) are variably adapted and insufficiently institutionalized at the national level. Addressing this gap requires NOCs and major sports organizations to put more efforts in embedding athletes’ welfare into their governance agendas, ensuring equity and inclusivity across all disciplines and performance levels. This calls for further development in this area, including a stronger link between evidence-based knowledge and practical implementation, a higher diversification of funding sources to sustain these activities, a wider exploitation of best practices, and the monitoring of organizational performance in assisting their elite athletes.

The survey results revealed also encouraging trends, showing examples of good practices, particularly in countries like France, Spain, and Portugal, where comprehensive and long-term services exist. These models demonstrate the feasibility of athlete-centered frameworks when supported by adequate resources, multi-sectoral collaboration, and formalized policies (35, 73, 74). The integration of educational institutions, mental health services, and career development support reflects the recognition of the centrality of inter-institutional partnerships and a multi-sectorial approach. Such collaborations resonate with sustainability principles, emphasizing systemic equity, accountability, and resource durability (19).

Digital innovation also emerged from respondents’ comments as a promising avenue to overcome structural and resource-related barriers. Online platforms, mobile applications, and e-counselling tools can provide scalable, cost-effective support, extending services to athletes regardless of geography or competitive status (57, 58). In this framework, European policy initiatives and ERASMUS + Sport Collaborative Partnerships, such as the PORTAL project, could represent a crucial avenue of integration of digital resources and traditional support structures by offering useful tools for mental health screening, vocational guidance, peer communities, and real-time support (60). These tools align with the growing emphasis on inclusivity, accessibility, and lifelong learning at European level (59), and may help mitigating inequities in access to services.

Overall, the present findings confirm the urgent need to situate athletes’ career transitions within a sustainability paradigm. Retirement should not be a source of disadvantage but an opportunity for reintegration and growth (24, 26). This requires moving beyond *ad hoc* initiatives towards systemic, long-term strategies that embed career assistance, mental health care, and practical support throughout the athletic lifecycle (18, 28, 64). Multi-level governance, adequate funding, and cross-sectoral collaboration are essential to institutionalize these frameworks, ensuring equity, and harness athletes’ human and social capital as resources for the society at large. In this sense, NOCs and international sports organizations have a dual responsibility: to safeguard the welfare of athletes as a matter of rights and to secure the sustainability of sport in line with the United Nations Sustainable Development Goals (4, 5).

## 5 Conclusions

The results of the present study show a fragmented landscape of the support services provided to top-performance athletes across various European countries. While encouraging efforts are evident in the domains of mental health, career transitions, and additional services, the overall picture reveals significant disparities in service provision, structure, access, and awareness, which hinder the development of holistic, athlete-centered support systems. The present results must be interpreted considering the broader literature (60) and policy frameworks, which emphasize sustainability, good governance, and the safeguarding of athletes’ rights throughout their careers and beyond (3, 53). From a practical perspective, the findings suggest that NOCs and major sport organizations should move towards institutionalizing comprehensive, long-term support frameworks, accompanying elite athletes throughout their careers. First, systematic needs assessments should be embedded to tailor services, ensuring that interventions are evidence-based and equitable rather than restricted to medal-winning athletes (23). Second, the integration of dual career pathways with education and vocational training must be expanded, as these strategies represent one of the most effective buffers against the psychological and social risks of career termination (38, 44, 75, 76). Furthermore, financial literacy and education services should be implemented to reduce potential financial burdens and instability in the life after the elite sport career (47, 48). Third, investment in accessible and culturally sensitive mental health services is critical, addressing persistent stigma and ensuring early prevention (27, 47). Fourth, NOCs should adopt and scale digital tools, offering cost-effective opportunities to wider athletic population, investing in inclusivity, and real-time access to services (57, 58). Finally, sustainable governance models should emphasize elite athletes’ welfare as a dimension of organizational accountability and good governance, embedding support systems within national action plans and aligning with broader sustainability goals. By institutionalizing these measures, sport organizations can ensure that retirement becomes a structured and empowering process, reinforcing both athlete well-being and the long-term sustainability of elite sport systems.

Overall, several recommendations could be derived from the present findings, which might be summarized as follows:

1. Countries should establish national regulatory frameworks for athlete support services, including minimum standards for mental health, career transition, and life assistance programs;
2. Countries should ensure equitable access to services across all elite athletes’ career paths, strengthening a developmental approach rather than a sporting outcomes-based one (e.g., supporting elite athletes during their athletic journey and not just considering them upon winning a medal), with special attention to youth athletes and those in early or transitional phases of their careers;
3. Countries should strengthen inter-institutional partnerships, particularly with mental health providers, educational institutions, career agencies, and companies to support dual careers and long-term professional and personal development in and out of elite sport;
4. Countries should develop centralized communication strategies to actively disseminate information about services, improve athletes’ awareness, and reduce stigma, especially around mental health;
5. Countries should implement and standardize assessment tools to identify and track athlete needs throughout their careers, enabling personalized and timely support;
6. Countries should diversify funding models, including stronger public-private collaboration and optimized use of Olympic Solidarity and EU programs;
7. Countries should consider the athletes’ voices through structured feedback mechanisms to refine service quality, relevance, and delivery methods.

Whilst promising practices are emerging, systemic efforts are necessary to ensure that support services are not a privilege tied to performance, but a right accessible to all elite athletes committed to excellence in sport and life. In this regards, the growing digital innovation efforts may help overcoming these obstacles, by creating content and services accessible to a wider elite athletes’ population. Digital tools can integrate multiple support services ensuring cost-effective solutions to address the intricate challenges athletes face during career termination. In this framework, the PORTAL project's platform would represent a useful and effective tool in translating evidence-based information into a comprehensive, digital solution designed for long-term sustainability.

To note, this study focused only on the opinions of members of sport organizations. To track the quality and effectiveness of specific initiatives, to offer a deeper understanding of the current challenges and opportunities, and to formulate sustainable possible implementation of career assistance practices within the sport ecosystem, further research is strongly needed. Furthermore, these findings should be compared with the perceptions of elite athletes on available support services to align real needs to implementation strategies.

The present study must be considered in light of several limitations. Firstly, accurateness of findings is challenged by the lack of information from many European NOCs declining to participate. Although our sample might be considered representative of different career assistance approaches within the European Union (22, 56), a higher number of participant respondents should be envisioned in future studies to provide a more comprehensive picture of mental health, career transition and assistance services. Furthermore, future research is needed to assess the progress of ongoing development of National and/or organizational regulatory frameworks and services provision within European contexts. Secondly, future studies should involve samples composed by International (e.g., International Sports Federations) and National sports bodies (e.g., NOCs, National Sports Federations) confronting different sources of information, approaches and initiatives in this field. Thirdly, the data collection performed in the present study (e.g., semi-structured electronic survey) could have influenced the depth of the collected information. Thus, mixed-method and triangulating study designs (e.g., integrating quantitative and qualitative data collected through survey responses, in-depth interviews, and/or public/institutional data) should be envisioned in future studies. Such research methods could also integrate culturally sensitive approaches to better understand cultural differences determining and/or affecting career assistance practices within diverse national and sports settings.

## Data Availability

The original contributions presented in the study are included in the article/Supplementary Material, further inquiries can be directed to the corresponding authors.
