# Supplementary material for: Sustainability of top-performance athletes’ mental health and career retirement support services: European major sports organizations’ perspective
Source: Front Sports Act Living. 2026 Apr 1;8:1722556. doi: 10.3389/fspor.2026.1722556 (PMC13079695; doi:10.3389/fspor.2026.1722556)
Supplement: Supplementary file 1 [file supplementaryfile1.docx]

**Supplementary File S1 - Structure of the tailored electronic survey**

**Accompanying letter:**

Dear Participant

The present survey is part of a research carried under the Erasmus+ Sport European funded project “SupPorting OlympiAns Transitioning to Real Life in concern of their mental health - PORTAL” (Project ID: 101184857) aimed to support Olympians and high-performance athletes during their transition to post-retirement life.

The project's core objective is to develop and implement an inclusive online platform, complemented by a network of “RealLife Transition Officers”, to provide comprehensive support to elite athletes, particularly those facing fewer opportunities.

In this framework, the present survey has been designed to map the policies, programmes, and practices surrounding the mental health, career transition, and support services for high performance athletes within European sports organizations.

By filling in the present survey, your contribution will help shaping a deeper understanding of the state of art of the multilevel support towards top-performance athletes within the European context, which will be crucial to address critical areas of intervention to promote better conditions and sustainability of career transitions out of sport and mental health support for elite athletes.

The survey includes both closed-ended and open-ended questions, organized in the following sections:

- Mental Health Services

- Career Transitions Services

- Other Support Services

- Good Practices and Challenges/needs in relation to Support Services

Please answer the questions to the best of your ability, and where possible, include references, links, or any additional resources that can substantiate your responses.

The Survey will take about 10-15 minutes to be completed.

Please consider that there are no right or wrong answers to the proposed questions, that your responses are confidential, and that you can drop from the Desk Research survey at any time and for any reason. Your responses will be anonymously electronically archived for data analysis.

By answering the survey, you agree to contribute in the research and analysis. If you do not think your contribution is possible at this stage, we would still like to thank you for considering the possibility and for reading this request.

Should you have questions, please do not hesitate to contact us.

Research unit within the PORTAL Project

San Raffaele Open University of Rome

Contact person:

Dr Flavia Guidotti, PhD

flavia.guidotti@uniroma5.it

Agreement to fill in this Desk Research Survey*

 Yes

 NO

**Section 1: General Information**

Please provide general information regarding your Organization:

- Country: [short text]

- Name of the Organization: [short text]

- Is your organization a National Olympic Committee?

 Yes

 NO

If not, please specify. [short text]

- Please provide additional information on the size of your organization:

- indicate the number of members: [short text]

- indicate the number of employees: [short text]

- What is your role/position within the Organization (for example, senior manager, middle manager, project manager, counsellor, etc.)? [short text]

- Is your organization active in supporting top-performance athletes' career transitions?

 Yes

 NO

 In development

- Is your organization active in supporting top-performance athletes' career retirement?

 Yes

 NO

 In development

- Is your organization active in providing mental health support services for top-performance athletes?

 Yes

 NO

 In development

- Is your organization active in providing additional support services for top-performance athletes?

 Yes

 NO

 In development

****************************************************

**Section 2: Mental Health Support Services**

Please answer the questions to the best of your knowledge. When possible, please deepen your answers to closed-ended items including additional information within open comments space.

1. Does your organization provide mental health resources, services or support for high-performance athletes?

 Yes

 NO

 In development

2. If yes, what type of mental health support services are available to high-performance athletes? (Please check all that apply)

o In-house sports psychologists or counsellors

o Access to external mental health professionals

o Peer support programmes

o Mental health awareness training for athletes, coaches and staff

o Crisis intervention services

o Other…

3. If available, to which athletes (competition level) are mental health support services offered? (Please check all that apply)

o European medalist athletes

o World medalist athletes

o Olympic medalist athletes

o Other…

Please, integrate your answers to question 1, 2 and 3 by providing additional details and/or information regarding mental health support practices in place. [Open comment]

4. What are the most common mental health challenges faced by your top-performance athletes, especially during the retirement period? Please provide your perceptions/opinions. [Open comment]

5. Does your organization assess the mental health needs of top-performance athletes?

 Yes

 NO

 In development

6. How does your organization assess the mental health needs of top-performance athletes? (Please check all that apply)

o Regular check-ins with coaching staff

o Anonymous surveys or assessments

o Individual counselling sessions

o Athlete self-referral process

o No assessment practices are in place

o Other…

Please, integrate your answers to question 4, 5 and 6 by providing additional details and/or information regarding the practices in place. [Open comment]

7. Who finances mental health services for top-performance athletes in your organization? (Please check all that apply)

o Public funds

o Private sector

o Membership fees

o Own funds

o Other…

If possible, please deepen your answer to question 7 by providing additional details/information [Open comment]

8. What are the main obstacles to accessing mental health services for athletes in your organization? (Please check all that apply)

o Stigma

o Lack of funding

o Lack of professionals involved

o Lack of available services

o Other…

If possible, please deepen your answer to question 8 by providing additional details/information [Open comment]

9. How do you perceive high performance athletes' awareness regarding their mental health needs? [Scale]

Not aware at all ➊➋➌➍➎➏➐➑➒➓ Highly aware

10. How effective do you believe the available mental health services are in supporting high performance athletes?

o Very effective

o Somewhat effective

o Not very effective

o Not effective at all

o Unsure

11. Are mental health services in supporting high performance athletes provided independently from the athletes' sport discipline?

o Yes, services are available to both Individual and Team sports athletes

o No, services are available mostly to Individual sports athletes

o No, services are available mostly to Team sports athletes

o No, there aren't services available

o Unsure

If you have further comments regarding mental health support services in place in your organization, please use this space. [Open comment]

If you have links/references to mental health programmes, services and/or policies in place in your organization to complement your answers please use this space. [Open comment]

****************************************************

**Section 3: Career Transition Support Services**

Please answer the questions to the best of your knowledge. When possible, please deepen your answers to closed-ended items including additional information within open comments space.

12. Does your organization offer career transition support services for high performance athletes moving from professional sports to other career paths?

 Yes

 NO

 In development

13. If yes, what types of career transition services are provided? (Please check all that apply)

o Career counselling services

o Job placement services

o Competences/Skills development programs

o Competences/Skills assessment/monitoring tools

o Networking opportunities with industry professionals

o Educational workshops (e.g., resume building, interview skills)

o Financial planning and management support

o Other…

14. If available, to which athletes (competition level) are support services offered? (Please check all that apply)

o European medalist athletes

o World medalist athletes

o Olympic medalist athletes

o Other…

Please, integrate your answers to question 12, 13 and 14 by providing additional details and/or information regarding career transitions support services and practices in place. [Open comment]

15. Do high performance athletes have access to ongoing career transition support services after they retire from competitive sports?

 Yes

 No

 Sometimes, depending on individual needs

16. How long after retiring from sports do athletes have access to career transition services?

o up to 6 months

o up to 1 year

o up to 2 years

o Unlimited

o No services are available

o Other…

17. How long does the post-sport career transition support last?

o up to 6 months

o up to 1 year

o up to 2 years

o Unlimited

o No services are available

o Other…

Please, integrate your answers to question 15, 16, and 17 by providing additional details and/or information regarding support services availability, duration of support, and service provision's characteristics. [Open comment]

18. How do you perceive high performance athletes' awareness regarding their career transition needs? [Scale]

Not aware at all ➊➋➌➍➎➏➐➑➒➓ Highly aware

19. How effective do you believe the available career transition services are in supporting high performance athletes during the retirement period?

o Very effective

o Somewhat effective

o Not very effective

o Not effective at all

o Unsure

20. Are mental health services in supporting high performance athletes provided independently from the athletes' sport discipline?

o Yes, services are available to both Individual and Team sports athletes

o No, services are available mostly to Individual sports athletes

o No, services are available mostly to Team sports athletes

o No, there aren't services available

o Unsure

If you have further comments regarding career transition support services in place in your organization, please use this space. [Open comment]

If you have links/references to career transition programmes, services and/or policies in place in your organization to complement your answers please use this space. [Open comment]

****************************************************

**Section 4: Other Support Services**

Please answer the questions to the best of your knowledge. When possible, please deepen your answers to closed-ended items including additional information within open comments space.

21. What types of additional support services are available to high-performance athletes in your organization? (Please check all that apply)

o Housing assistance

o Financial aid and budgeting support

o Legal or contractual assistance

o Family support services

o Nutrition counselling

o Travel and logistics support

o Social support services (e.g., community building, social networks)

o Other…

22. If available, to which athletes (competition level) are mental health support services offered? (Please check all that apply)

o European medalist athletes

o World medalist athletes

o Olympic medalist athletes

o Other…

23. How do high performance athletes access these support services? (Please check all that apply)

o Directly through the organization’s internal support network

o Via referrals from coaches or staff

o Self-referral process

o Other…

Please, integrate your answers to question 21, 22 and 23 by providing additional details and/or information regarding support services availability, duration of support, and service provision's characteristics. [Open comment]

24. How do you perceive high performance athletes' awareness regarding their support service’s needs? [Scale]

Not aware at all ➊➋➌➍➎➏➐➑➒➓ Highly aware

25. How effective do you believe the available additional support services are in helping high performance athletes manage both their athletic and personal lives and in the retirement period?

o Very effective

o Somewhat effective

o Not very effective

o Not effective at all

o Unsure

26. Are mental health services in supporting high performance athletes provided independently from the athletes' sport discipline?

o Yes, services are available to both Individual and Team sports athletes

o No, services are available mostly to Individual sports athletes

o No, services are available mostly to Team sports athletes

o No, there aren't services available

o Unsure

If you have further comments regarding additional services in place in your organization, please use this space. [Open comment]

If you have links/references to additional programmes, services and/or policies in place in your organization to complement your answers please use this space. [Open comment]

****************************************************

**Section 5: Challenges and Opportunities**

Please answer the questions to the best of your knowledge. When possible, please deepen your answers to closed-ended items including additional information within open comments space.

27. Are athletes informed about the existence of the available services?

o Yes, we actively promote them

o Sometimes we mention them

o No, most athletes are unaware

28. How many athletes use the available services?

o Less than 25%

o 25-50%

o 50-75%

o 75% or more

Please, integrate your answers to question 27 and 28 by providing additional details and/or information. [Open comment]

29. Is there a regulatory framework at your organization explicitly defining top-performance athletes' career assistance practices?

 Yes

 No

 Unsure

30. In absence of a regulatory framework at your organization explicitly defining top-performance athletes' career assistance practices, are there temporary initiatives?

 Yes

 No

 Unsure

Please, integrate your answers to question 29 and 30 by providing additional details and/or information. [Open comment]

31. What are the main challenges your organization faces in supporting high performance athletes? [Open comment]

32. What improvements or additional support services do you think would most benefit high performance athletes in your organization? [Open comment]

33. What partnerships or collaborations (e.g., with educational institutions, mental health organizations, etc.) do you think would be beneficial for enhancing high performance athletes support services? [Open comment]

34. Any additional comments or insights on supporting mental health, career transition, transition out of sport for high-performance athletes? [Open comment]

****************************************************

**Conclusion**

Thank you for participating in this survey.

Your responses will provide valuable insights for improving the quality of our Desk Research data collection towards the Project PORTAL implementation!
